# Documenting Environmentally Acquired *Mycobacterium intracellulare* subsp. *chimaera* Pulmonary Disease Soon After Bronchiectasis Onset

**DOI:** 10.1093/cid/ciag015

**Published:** 2026-01-10

**Authors:** Jennifer R Honda, Rachel N Wilsey, Chelsea K Raulerson, Liang-Hao Ding, Christian L Castaneda, James L Joyner

**Affiliations:** Department of Cellular and Molecular Biology, School of Medicine, University of Texas Health Science Center at Tyler, Tyler, Texas USA; Department of Cellular and Molecular Biology, School of Medicine, University of Texas Health Science Center at Tyler, Tyler, Texas USA; Department of Cellular and Molecular Biology, School of Medicine, University of Texas Health Science Center at Tyler, Tyler, Texas USA; Department of Cellular and Molecular Biology, School of Medicine, University of Texas Health Science Center at Tyler, Tyler, Texas USA; Department of Medicine, John A. Burns School of Medicine, University of Hawai’i, Honolulu, Hawai'i USA; Hawai’i Infectious Disease Associates, Honolulu, Hawai'i USA

**Keywords:** *mycobacterium intracellulare* subsp. *chimaera*, whole genome sequencing, environment, bronchiectasis, Hawai’i

## Abstract

It's difficult to prove exactly when and how people with bronchiectasis acquire nontuberculous mycobacteria (NTM) from the environment. We present data showing long-term household exposures of a homeowner and development of NTM pulmonary disease following the onset of bronchiectasis and correlating disease onset with longitudinal environmental sampling in an NTM geographic hotspot.

Households are established sources for nontuberculous mycobacteria (NTM) exposures. However, documenting NTM infection events and establishing links between environmentally acquired NTM pulmonary disease *a priori* for people with bronchiectasis is challenging. Herein we applied a 12-year environmental sampling campaign of a single home in the NTM geographic hotspot of Hawai’i to monitor the diversity and stability of environmental NTM over time [1, 2]. Coincidentally, 11 years into sampling, the resident was diagnosed with NTM pulmonary disease after developing bronchiectasis symptoms. Whole genome sequencing phylogenomic analyses of the patient's respiratory *Mycobacterium intracellulare* subsp*. chimaera* and longitudinally collected residential environmental isolates of the same species support the high probability of multiple household sources of exposure across time, but disease onset only after developing pre-disposing bronchiectasis. *Mycobacterium intracellulare* subsp*. chimaera* is a pulmonary disease causing NTM within the *Mycobacterium avium* complex, brought into recent spotlight due to its contamination of heater-cooler units (HCU) responsible for global outbreaks [3, 4] and is the most dominant NTM in environmental and respiratory specimens from Hawai`i [1].

## CASE PRESENTATION

**Figure 1.**
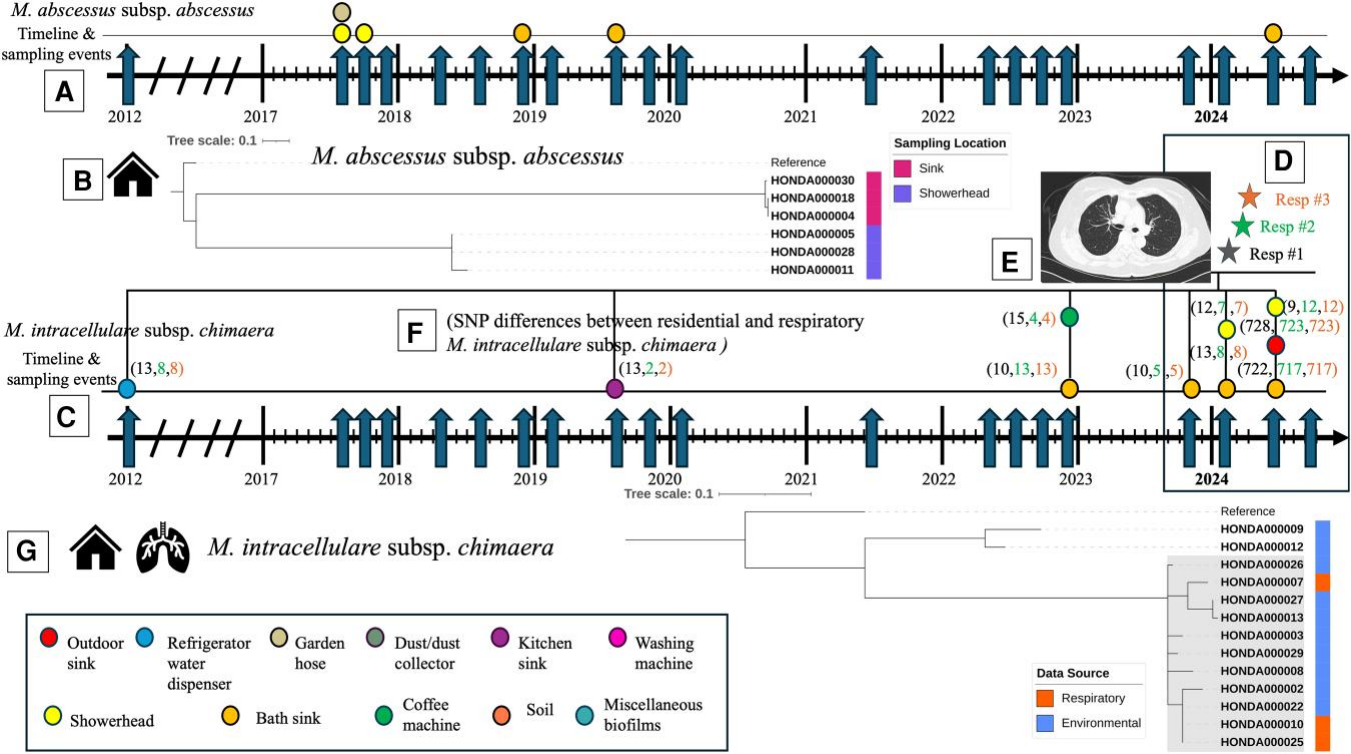
Longitudinal environmental sampling event outcomes with comparative phylogenomic analyses showcasing *M. abscessus* subsp. *abscessus* in the environment, but high likelihood of acquisition of residential *M. intracellulare* subsp. *chimaera* by the homeowner upon a new bronchiectasis diagnosis. *A*, Environmental niches that were culture positive for *M. abscessus* subsp. *abscessus* over the 12-year sampling period. *B*, Phylogenetic tree of *M. abscessus* subsp. *abscessus* isolates cultured from the residence, including isolates from a sink and showerhead sources within the same home. Scale bar = estimated genetic distance between isolates after 1000 bootstraps. *C*, Circles indicate the niches that were culture positive for *M. intracellulare* subsp. *chimaera* over the sampling period. *D*, Box: Indicates that in 2024, the homeowner developed *M. intracellulare* subsp. *chimaera* lung disease after bronchiectasis onset. From 3 sequential sputum samples, 3 *M. intracellulare* subsp. *chimaera* isolates (Resp nos. 1–3) were identified. *E*, CT scan showing waxing and waning lung nodules measuring up to 12 mm within the right lower lobe. Stable bronchiectasis and mucous plugging. *F*, All residential and respiratory *M. intracellulare* subsp. *chimaera* were whole genome sequenced and SNP differences between each respiratory isolate and *M. chimaera* recovered from each residential niche were determined and indicated as (“X, Y, Z”). (Values correspond to SNP differences between “Resp#1, Resp#2, Resp #3” isolates respectively and the indicated environmentally recovered isolate). SNP counts <38 were deemed highly similar. *G*, Phylogenetic tree of *M. intracellulare* subsp. *chimaera* isolates, pruned to show a representative isolate from each sampling event. Patient sputum isolates and residential isolates are indicated. The highlighted clade indicates environmental isolates matching the 3 respiratory samples by pairwise SNP distance. Scale bar = estimated genetic distance between isolates after 1000 bootstraps. Abbreviations: CT, computed tomography; NTM, nontuberculous mycobacteria; SNP, single-nucleotide polymorphism. **Box:** Key for the circles corresponding to the different environmental niches sampled from the home.

A 79 year-old female living her entire life in Hawai`i volunteered her home a decade earlier for routine environmental NTM sampling to investigate NTM changes in common household niches over an extended period. She had a history of stage 1b breast cancer post lumpectomy and radiation therapy in 2014 and was referred to the pulmonary clinic in 2022 for pulmonary nodules. Her chest computed tomography (CT) showed bronchiectasis (Figure 1*E*). Her nodules resolved on radiologic follow up with no intervention. She returned for reevaluation in May 2024 with daily cough productive of thick sputum and dyspnea on exertion with the ability to perform only 2–3 metabolic equivalents of task. Although previously there were no functional limitations, wheelchair assistance was required with prolonged ambulation with an unexplained 20-pound weight loss. Sputum specimens were ordered; of which 1 of 3 stained 1 + acid-fast bacilli and grew *Mycobacterium intracellulare* subsp*. chimaera* and 1 of 3 was acid-fast bacilli negative but grew *Mycobacterium intracellulare* subsp. *chimaera*. Repeat chest CT demonstrated no nodules and stable bronchiectasis. After infectious disease referral, 1 of 3 sputum samples again grew *Mycobacterium intracellulare* subsp. *chimaera*. Because the patient met the diagnostic criteria for NTM pulmonary disease [5], azithromycin 500 mg and ethambutol 1600 mg every M/W/F was started in September 2024 and returning in October 2024 with near complete cough resolution and improved appetite. After considering drug interactions and medication side effects, rifampin 600 mg M/W/F was added in November 2024. At that time, she was unable to expectorate any sputum and was judged to be “culture negative” from a practical standpoint. Her pulmonary stamina improved, became wheelchair independent, made travel plans, and stopped losing weight. The patient continued to do well with frequent follow-up finishing her treatment a year later.

## METHODS

Between 2012 and 2024, a single volunteer collected 155 environmental samples from the patient's home on O`ahu, Hawai`i across 20 independent sampling events. Environmental samples were cultured for viable NTM using standard microbiological approaches [1, 2], genomic DNA was extracted, and preliminary identification of NTM was performed using partial *rpoB* gene sequencing [1, 2, 6]. Subsequently, clinically relevant DNA from residential and patient respiratory *Mycobacterium intracellulare* subsp. *chimaera* and *M. abscessus* subsp. *abscessus* were subjected to acid-fast bacilli smear microscopy and whole genome sequencing (WGS). The University of Texas, Tyler Institutional Review Board (IRB 2025-107) provided ethical review.

Illumina Nextera XT, DNA Flex, or DNA Prep sequencing libraries were constructed and sequenced on the MiSeq, HiSeq, NextSeq, or NovaSeq (Illumina, San Diego, California, USA) using paired end 2 × 300 bp or 2 × 250 bp for 40 × coverage per genome. Reads were trimmed using SeqPurge (2020_03) and evaluated for quality using FastQC (v0.12.1) and contamination using kraken2 (version 2.1.3, downloaded May 2024) [7]. After quality checks, WGS data set contained 24 *Mycobacterium intracellulare* subsp. *chimaera* and 6 *M. abscessus* subsp. *abscessus* isolates. To identify single nucleotide polymorphisms (SNPs), snippy and snippy-core (version 4.6.0) were used with CDC2015-22-71 (GCF_002166795.1) and ATCC19977 (GCF_000069185.1) as *Mycobacterium intracellulare* subsp. *chimaera* and *M. abscessus* subsp. *abscessus* references, respectively. Pairwise distance between respiratory and residential isolates were computed using snp-dists (version 0.8.2). Cleaned SNPs from snippy-core full alignment, with recombination removed via gubbins (version 3.4) [8], were input into raxmlHPC (8.2.12) to build phylogenetic trees using 1000 bootstraps and visualized using iTOL [9].

To determine whether *M. abscessus* subsp. *abscessus* isolates belonged to dominant circulating clones (DCC), consensus fastas generated by snippy combined with Ruis *et al* data [10] were used to construct a phylogenetic tree. Parsnp (version 2.1.3) [11] aligned core genomes, gubbins removed recombination (version 3.4) [8], and the final tree was generated by raxmlHPC (8.2.12) [12] using GTRCAT.

## RESULTS

Of environmental samples collected, 30% (47/155) cultured NTM. Overall, 17 unique NTM species were identified, and no one species predominated the collection. Of relevance, *M. abscessus* subsp. *abscessus* and *Mycobacterium intracellulare* subsp. *chimaera* comprised 9% and 19%, respectively, of all species recovered.

The residence yielded multiple isolates of *M. abscessus* subsp. *abscessus* across several years (Figure 1*A*) encompassing two groups of matching samples stratified according to location (showerhead vs sink; Figure 1*B*). Of these, the showerhead group was more closely related to the *M. abscessus* ATCC 19977 reference genome (median SNP distance 38 vs 342; Supplementary Table 1); nevertheless, both groups clustered within DCC1.

The residence also produced multiple isolates of *Mycobacterium intracellulare* subsp. *chimaera* (Figure 1*C*). Since the homeowner's sputum samples harbored *Mycobacterium intracellulare* subsp. *chimaera,* pairwise SNP distances between residential and respiratory isolates (Figure 1*D*) were computed. Using previously reported thresholds of <20 SNPs indicative of a match [13] and <38 SNPs being suggestive of a match, pairs were sorted for likely, possible, and non matches; when multiple isolates originated from one sample, a representative isolate was chosen. All 3 respiratory *Mycobacterium intracellulare* subsp. *chimaera* isolates matched each other, with ≤11 SNPs. Notably, the first recovered residential *Mycobacterium intracellulare* subsp. *chimaera* from the refrigerator water dispenser in 2012 matched with respiratory isolates obtained 12 years later (pairwise SNP distances:13, 8, 8) (Figures 1*D*, 1*F*; Supplementary Table 2). Finally, when pairwise SNP distances between residential and respiratory isolates were compared, 8 of 10 residential *Mycobacterium intracellulare* subsp. *chimaera* matched with each of the 3 respiratory isolates and formed a clade in the phylogenetic tree (Figure 1*G*).

## DISCUSSION

Linking environmental exposures directly to disease a patient develops later in life is difficult. Most studies performed to date are “after the fact” studies, identifying patients suspected or diagnosed with NTM pulmonary disease, then performing environmental studies to identify exposures. In other cases, environmental sampling is performed post-outbreaks to find environmental matches as root sources or environmental NTM are matched with respiratory NTM in non-patient matched studies. Yet there is a paucity of studies demonstrating environmental NTM exposures prior to acquisition of pulmonary disease.

Rather than traditionally focusing on patient samples first, our primary goal was to prioritize environmental NTM by documenting and tracing longitudinal NTM diversity changes in a home located in a geographic hotspot across a decade. We had no way to predict the homeowner would eventually be diagnosed with NTM pulmonary disease. Because of this serendipity, several novel findings were uncovered. First, our data showing residential *Mycobacterium intracellulare* subsp. *chimaera* recovery in 2012, intermittent isolation across time, and robust recovery in 2024 indicate long-term adaptation and stability of *Mycobacterium intracellulare* subsp. *chimaera* in a home. Second, identification of nearly identical *Mycobacterium intracellulare* subsp. *chimaera* from the patient's refrigerator, kitchen/bath sinks, coffee machine, and showerhead suggests multiple possible sources of home exposure. Third, and most importantly, WGS and comparative phylogenomic analyses support multiple residential-derived *Mycobacterium intracellulare* subsp. *chimaera* isolates matching to *Mycobacterium intracellulare* subsp. *chimaera* isolated from respiratory cultures including environmental samples collected before the patient developed symptoms. These indications along with the presence of known pre-disposing host factors (eg, age, female gender, Asian ethnicity) and clinical developments (eg, bronchiectasis), likely factored into the development of pulmonary disease. This study has limitations. First is the absence of NTM-negative sputum samples prior to bronchiectasis onset to rule out subclinical infection at an earlier timepoint. Yet disease diagnosis requires symptoms, positive cultures, and radiographic findings and in the absence of these, cultures would not be warranted. Second, the mutation rate of *Mycobacterium intracellulare* subsp. *chimaera* is not currently known and cannot be used to suggest a time for either colonization or new infection. Low SNP distances could equally indicate a long-standing, low-replicating population persisting in both the residence and the host rather than a fresh infection event. Nevertheless, this case study renews discussions to answer the long-standing question of which came first, bronchiectasis or NTM pulmonary disease by providing unique evidence of pervasive household exposures before the development of either bronchiectasis or NTM pulmonary disease.

## Supplementary Material

ciag015_Supplementary_Data

## Supplementary Data

Supplementary materials are available at *Clinical Infectious Diseases* online. Consisting of data provided by the authors to benefit the reader, the posted materials are not copyedited and are the sole responsibility of the authors, so questions or comments should be addressed to the corresponding author.

## Notes

***Acknowledgments***: Authors thank Stephanie Dawrs, Ravleen Virdi, Grant Norton, Edward Chan MD, Michael Strong PhD, Nabeeh Hasan PhD, Elaine Epperson PhD, and James Crooks PhD for prior project contributions. Diagnostics Laboratory Services, Inc. (Aiea, Hawai’i) provided clinical, de-identified isolates included in this study. Authors thank Dr Max Salfinger for critical review of this manuscript.

Conceptualization and study design: J. R. H.

Data acquisition: J. R. H., R. N. W., C. K. R., L.-H. D.

Provided CT scan: J. L. J.

Clinical presentation: C. L. C., J. L. J.

Data analyses: J. R. H., R. N. W., C. K. R., L.-H. D.

Drafted the manuscript: J. R. H., R. N. W., C. K. R., L.-H. D., C. L. C., J. L. J.

Edited final version of the manuscript: J. R. H., R. N. W., C. K. R., L.-H. D., C. L. C., J. L. J.

Provided funding: J. R. H.

Supervisory functions: J. R. H.

***Data availability statement***. The data underlying in this article are available at Bioproject X and can be accessed with accession numbers Y.

***Financial support*.** This work was funded, in part, by the National Science Foundation #1743587, Division of Environmental Biology, as part of the Ecology and Evolution of Infectious Diseases program. J. R. H. acknowledges support from the Padosi Foundation and the University of Texas Health Science Center at Tyler.
