# Supplementary material for: Documenting Environmentally Acquired Mycobacterium intracellulare subsp. chimaera Pulmonary Disease Soon After Bronchiectasis Onset
Source: Clin Infect Dis. 2026 Jan 10;82(6):e1322–5. doi: 10.1093/cid/ciag015 (PMC13341260; doi:10.1093/cid/ciag015)
Supplement: ciag015_Supplementary_Data [file ciag015_supplementary_data.pdf]

Supplemental Table 1. Pairwise distance matrix of *M. abscessus* subsp. *abscessus* isolates

	HONDA 000005	HONDA 000011	HONDA 000028	HONDA 000004	HONDA 000030	HONDA 000018	Reference
HONDA000005	0						
HONDA000011	7	0					
HONDA000028	0	7	0				
HONDA000004	352	359	352	0			
HONDA000030	351	358	351	1	0		
HONDA000018	352	359	352	0	1	0	
Reference	38	45	38	342	341	342	0

A pairwise distance matrix representing the number of SNPs were each isolate differs from every other isolate. The zeros at the end of each row represent the distance between each isolate and itself. The reference sample was Mycobacteroides abscessus ATCC19977 (GCF_000069185.1)

Supplemental Table 2. Pairwise distance matrix of *M. intracellulare* subsp. *chimaera* isolates

	HONDA 000008	HONDA 000022	HONDA 000013	HONDA 000019	HONDA 000002	HONDA 000006	HONDA 000024	HONDA 000014	HONDA 000029	HONDA 000001	HONDA 000021	HONDA 000027	HONDA 000012	HONDA 000009	**HONDA 000007**	**HONDA 000015**
HONDA000008	0															
HONDA000022	10	0														
HONDA000013	15	15	0													
HONDA000019	17	17	2	0												
HONDA000002	12	6	17	19	0											
HONDA000006	12	6	17	19	2	0										
HONDA000024	18	18	15	17	20	20	0									
HONDA000014	22	22	19	21	24	24	22	0								
HONDA000029	7	7	12	14	9	9	15	19	0							
HONDA000001	136	136	139	141	138	138	142	146	133	0						
HONDA000021	26	26	31	33	28	28	34	38	23	152	0					
HONDA000027	14	14	1	3	16	16	14	18	11	138	30	0				
HONDA000012	719	719	724	726	721	721	727	731	716	668	735	723	0			
HONDA000009	725	725	730	732	727	727	733	737	722	674	741	729	14	0		
**HONDA000007**	**13**	**13**	**10**	**12**	**15**	**15**	**13**	**17**	**10**	**137**	**29**	**9**	**722**	**728**	**0**	
**HONDA000015**	**14**	**14**	**11**	**13**	**16**	**16**	**14**	**18**	**11**	**138**	**30**	**10**	**723**	**729**	**1**	**0**
**HONDA000025**	**8**	**2**	**13**	**15**	**4**	**4**	**16**	**20**	**5**	**134**	**24**	**12**	**717**	**723**	**11**	**12**
**HONDA000020**	**22**	**16**	**27**	**29**	**18**	**18**	**30**	**34**	**19**	**148**	**38**	**26**	**731**	**737**	**25**	**26**
**HONDA000010**	**8**	**2**	**13**	**15**	**4**	**4**	**16**	**20**	**5**	**134**	**24**	**12**	**717**	**723**	**11**	**12**
HONDA000003	9	7	14	16	11	11	17	21	6	135	25	13	718	724	12	13
HONDA000017	14	14	19	21	16	16	22	26	11	132	30	18	716	722	17	18
HONDA000026	10	10	15	17	12	12	18	22	7	128	26	14	712	718	13	14
HONDA000016	718	718	723	725	720	720	726	730	715	667	734	722	1	13	721	722
HONDA000023	14	14	1	3	16	16	14	18	11	138	30	0	723	729	9	10
Reference	188	184	193	195	190	190	196	200	185	183	204	192	755	761	191	192

A pairwise distance matrix representing the number of SNPs were each isolate differs from every other isolate. The zeros at the end of each row represent the distance between each isolate and itself. Cells shown in bold indicate the sputum samples from the homeowner; all other isolates were recovered from the residence. The reference sample was *Mycobacterium intracellulare subsp. chimaera* CDC 2015-22-71 (GCF_002166795.1).

Supplemental Table 2. Pairwise distance matrix of *M. intracellulare* subsp. *chimaera* isolates

	**HONDA 000025**	**HONDA 000020**	**HONDA 000010**	HONDA 000003	HONDA 000017	HONDA 000026	HONDA 000016	HONDA 000023	Reference
HONDA000008									
HONDA000022									
HONDA000013									
HONDA000019									
HONDA000002									
HONDA000006									
HONDA000024									
HONDA000014									
HONDA000029									
HONDA000001									
HONDA000021									
HONDA000027									
HONDA000012									
HONDA000009									
**HONDA000007**									
**HONDA000015**									
**HONDA000025**	**0**								
**HONDA000020**	**14**	**0**							
**HONDA000010**	**0**	**14**	**0**						
HONDA000003	7	21	7	0					
HONDA000017	12	26	12	13	0				
HONDA000026	8	22	8	9	6	0			
HONDA000016	716	730	716	717	715	711	0		
HONDA000023	12	26	12	13	18	14	722	0	
Reference	186	200	186	185	189	185	754	192	0

A pairwise distance matrix representing the number of SNPs were each isolate differs from every other isolate. The zeros at the end of each row represent the distance between each isolate and itself. Cells shown in bold indicate the sputum samples from the homeowner; all other isolates were recovered from the residence. The reference sample was *Mycobacterium intracellulare subsp. chimaera* CDC 2015-22-71 (GCF_002166795.1).
